# Heart rhythm complexity analysis in patients with inferior ST-elevation myocardial infarction

**DOI:** 10.1038/s41598-023-41261-8

**Published:** 2023-11-27

**Authors:** Shu-Yu Tang, Hsi-Pin Ma, Chen Lin, Men-Tzung Lo, Lian-Yu Lin, Tsung-Yan Chen, Cho-Kai Wu, Jiun-Yang Chiang, Jen-Kuang Lee, Chi-Sheng Hung, Li-Yu Daisy Liu, Yu-Wei Chiu, Cheng-Hsuan Tsai, Yen-Tin Lin, Chung-Kang Peng, Yen-Hung Lin

**Affiliations:** 1 Department of Internal Medicine, National Taiwan University Hospital Yunlin Branch, Yunlin, Taiwan; 2 Department of Electrical Engineering, National Tsing Hua University, Hsinchu, Taiwan; 3 Department of Biomedical Sciences and Engineering, National Central University, No. 300, Zhongda Road, Taoyuan, Taiwan; 4 Department of Internal Medicine, National Taiwan University Hospital, Taipei, Taiwan; 5 Department of Internal Medicine, National Taiwan University Hospital Hsinchu Branch, Hsinchu, Taiwan; 6 Department of Agronomy, Biometry Division, National Taiwan University, Taipei, Taiwan; 7 Department of Computer Science and Engineering, Yuan Ze university, Taoyuan, Taiwan; 8 Cardiology Division of Cardiovascular Medical Center, Far Eastern Memorial Hospital, New Taipei City, Taiwan; 9 Department of Internal Medicine, Taoyuan General Hospital, Taoyuan, Taiwan; 10 Division of Interdisciplinary Medicine and Biotechnology, Beth Israel Deaconess Medical Center/Harvard Medical School, Boston, USA; 11 Department of Internal Medicine, Division of Cardiology, National Taiwan University Hospital, 7 Chung-Shan South Road, Taipei, Taiwan; 12 Department of Inderal Medicine, Division of Cardiology, Taoyuan General Hospital, 1492 Zhongshan Road, Taoyuan, 33004 Taiwan

**Keywords:** Myocardial infarction, Nonlinear phenomena, Computational biology and bioinformatics

## Abstract

Heart rhythm complexity (HRC), a subtype of heart rate variability (HRV), is an important tool to investigate cardiovascular disease. In this study, we aimed to analyze serial changes in HRV and HRC metrics in patients with inferior ST-elevation myocardial infarction (STEMI) within 1 year postinfarct and explore the association between HRC and postinfarct left ventricular (LV) systolic impairment. We prospectively enrolled 33 inferior STEMI patients and 74 control subjects and analyzed traditional linear HRV and HRC metrics in both groups, including detrended fluctuation analysis (DFA) and multiscale entropy (MSE). We also analyzed follow-up postinfarct echocardiography for 1 year. The STEMI group had significantly lower standard deviation of RR interval (SDNN), and DFAα2 within 7 days postinfarct (acute stage) comparing to control subjects. LF power was consistently higher in STEMI group during follow up. The MSE scale 5 was higher at acute stage comparing to control subjects and had a trend of decrease during 1-year postinfarct. The MSE area under scale 1–5 showed persistently lower than control subjects and progressively decreased during 1-year postinfarct. To predict long-term postinfarct LV systolic impairment, the slope between MSE scale 1 to 5 (slope 1–5) had the best predictive value. MSE slope 1–5 also increased the predictive ability of the linear HRV metrics in both the net reclassification index and integrated discrimination index models. In conclusion, HRC and LV contractility decreased 1 year postinfarct in inferior STEMI patients, and MSE slope 1–5 was a good predictor of postinfarct LV systolic impairment.

## Introduction

ST-segment elevation myocardial infarction (STEMI) is an important disease, leading to serious sequelae and even death. In the current era of primary percutaneous coronary intervention, the annual incidence of STEMI has decreased and the outcomes have improved^1,2^. The annual incidence of STEMI decreased to 50–77/100,000 persons after 2000, however it still increases the risk of heart failure, arrhythmia, and sudden cardiac death, causing a heavy disease burden. Many studies have investigated the survival predictors after myocardial infarction, among which left ventricular (LV) contractility has been shown to be an important independent prognostic predictor in postinfarction patients^3^.

Heart rhythm complexity (HRC), derived from heart rate variability (HRV), is a novel non-linear measurement based on a 24-h Holter ambulatory recording^4^. HRC estimates the change in “complexity” of a system^5^. Based on a hypothesis of “breakdown”, the complexity of a system decreases in a diseased status^6,7^, such as heart failure^8,9^ and primary hyperaldosteronism^10^. HRC, including multiscale entropy (MSE) and detrended fluctuation analysis (DFA), has been reported to have better predictive power in many cardiovascular diseases compared to traditional HRV measurements^8,9^. In our previous study, we showed that anterior STEMI patients had depressed HRV and HRC^11^. The association between depressed HRV and renin-angiotensin system (RAS) has been demonstrated before^10^, and RAS activates rapidly after acute myocardial infarction^12^. RAS activation may contribute to postinfarct depressed HRV. HRV and HRC per se have been associated with the prognosis in postinfarction patients^13,14^, however the association of HRV and HRC with LV contractility is uncertain. Postinfarct LV systolic impairment can lead to further heart failure and sudden cardiac death. In the current era of coronary intervention, postinfarct survival has improved dramatically, however postinfarct LV systolic impairment is still an important issue. Many studies have investigated the use of HRV metrics to predict postinfarct survival, but few studies have investigated the association between HRV/HRC metrics and postinfarct LV contractility.

Even though many HRV studies have investigated anterior STEMI, few have focused on inferior STEMI. Inferior STEMI may provoke Bezold-Jarisch (BJ) reflex, causing hypotension, marked sinus bradycardia or high degree atrioventricular block^15–17^, and BJ reflex may influence HRV. Since HRV studies focusing on patients with inferior STEMI are rare, the aim of this study was to analyze the dynamic changes in postinfarct HRV/HRC metrics in patients with inferior STEMI and investigate the association between HRV/HRC and postinfarct LV systolic impairment.

## Methods and subjects

### Study participants

In this prospective study, we enrolled 33 inferior STEMI patients and 74 control. The inclusion criteria of inferior STEMI patients were: (1) patients with ST elevation in inferior leads without BJ reflex (severe bradycardia with hypotension), atrial fibrillation, or high degree atrioventricular block on ECG; (2) patients received successful revascularization; (3) patients agree to participate in this study. The exclusion criteria of the inferior STEMI patients were patients with chronic atrial fibrillation, sick sinus syndrome or high degree atrioventricular block. The inclusion criteria of control were: (1) patients with documented patent coronary artery or non-significant coronary artery disease (2) patients agree to participate in this study. The exclusion criteria of control were: (1) patients with a history of chronic atrial fibrillation, sick sinus syndrome, high degree atrioventricular block, heart failure, previous myocardial infarction, significant coronary artery disease or peripheral artery disease to avoid possible pathological influences in control subjects.

All control subjects had a 24-h Holter recording on the day before the coronary angiogram, and an echocardiogram during hospitalization. All inferior STEMI patients underwent a primary percutaneous coronary intervention within 12 h of symptom onset, and both echocardiography and Holter recording during the acute event (72 h within STEMI), and then 3 months, 6 months and 1-year postinfarct.

The study was approved by the Institutional Review Board of National Taiwan University Hospital (NTUH 104-S2696) for clinical research. All participants were informed of the study protocol before enrollment, and all signed informed consent forms which were stored in the hospital’s database. All methods were performed in accordance with the relevant guidelines and regulations.

### Echocardiography

All of the patients underwent standard transthoracic echocardiography (iE33 xMATRIX Echocardiography System, Philips, Amsterdam, Netherlands) based on the American Society of Echocardiography guidelines^18^. The left ventricular ejection fraction (LVEF) was measured on an apical 4-chamber view using the area-length method when there were regional wall abnormalities, or M-mode on a parasternal long axis view when there were no regional wall abnormalities^18,19^.

### Holter and data pre-processing

All of the patients maintained their original daily activities while undergoing 24-h ambulatory ECG recording (Zymed DigiTrak Plus/XT 24-Hour Holter Monitor Recorder, Philips, Amsterdam, Netherlands). The sampling rate of ECG recording was set at 250 Hz. Two experienced technicians inspected ECG strips to ensure accurate RR intervals and exclude any ectopic heartbeats. All of the patients completed a full ECG recording for at least 20 h. Four-hour segments of ECG data within the daytime (9 am to 6 pm) when the patient was awake were selected to avoid possible influences of the circadian cycle^20^. Any sudden increase in heart rate exceeding 40 bpm within 1 min was excluded from analysis because of the potential influence of heavy physical activity^21^. The data were processed automatically using MATLAB software^22,23^. To eliminate the spurious outlier or ectopic beats that could compromise the HRV/HRC analysis^24^, a two-step process was implemented. Initially, ectopic beats were identified and subsequently substituted with the interpolated value of the previous and preceded adjacent RR intervals. Then, outliers were also rectified using a moving filtering with a 40-beat window to identify RR intervals that deviated from the median by more than 2.5 times the standard deviation of the RR intervals in the window^25^. The RR intervals derived from the selected 4- hour data were divided into non-overlapping 5-min segments and the linear indices of those segments were calculated. These indices were then averaged to derive ensemble HRV parameters, serving as representative measures for each individual patient.

### Linear analysis

Based on the guidelines developed by the Task Force of the European Society of Cardiology^4^, we calculated linear HRV analysis including time domain and frequency domain indices. Time domain measures the variability of consecutive normal sinus heartbeats, and time domain indices include mean RR interval (mean RR), the standard deviation of RR intervals (SDNN), and the percentage of successive differences in RR intervals exceeding 20 ms (pNN_20_) and 50 ms (pNN_50_). Using fast Fourier transformation, RR intervals were transformed into frequency ranges. Signals were separated into several components by different frequency ranges, and high frequency (0.15–0.4 Hz; HF), and low frequency (0.04–0.15 Hz; LF) were calculated. Traditional linear HRV metrics are regulate by the autonomic nervous system. LF power reflects both sympathetic and parasympathetic tones, while HF power reflects parasympathetic modulation mostly.

### Non-linear measurements

Both DFA and MSE were used to estimate the intrinsic inter-beat similarity and complexity. DFA quantifies the intrinsic multifractality and self-similarity at different scales in a dynamic system^26,27^. By using slope exponents (α exponent) in a log–log plot to represent the fractal correlation of time series, we measured α1 (4–11 beats) and α2 (11–64 beats) exponents to represent behavior in short-term and long-term time scales, respectively. Crossover has been reported between short- and long-term exponents in both ill and healthy subjects^27^. Both short-term and long-term scale exponents were used in this study to describe fractal behavior in a physiological system.

**Figure 1:**
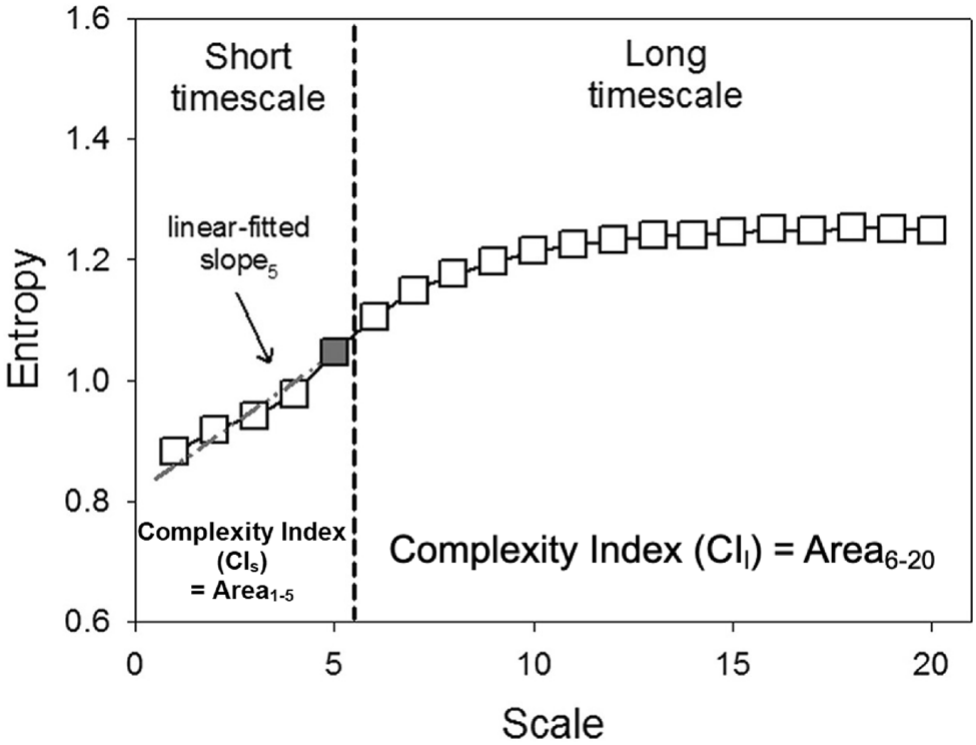
Quantification of MSE.

MSE analysis measures the complexity of an intrinsic system in a dynamic time series. Entropy estimation measures regularity on a single time scale; MSE uses the sample entropy (SampEn) algorithm to calculate an entropy value along with a “coarse graining” process to provide more information of system complexity in different time scales in a dynamic system^5,28^. However, the MSE might be underestimated due to the suboptimal filtering effects of 'coarse graining,' especially in the presence of high-frequency interference (e.g. ectopic beats or outliers) and nonstationary trends or oscillations^29,30^. To counteract the effects of nonstationarity, we not only removed outlier or ectopic beats but also eliminated oscillations slower than the very low frequency (VLF) range from the R-R interval series using empirical mode decomposition as an adaptive filter. In MSE analysis, we quantified four metrics: the SampEn value at scale 5 (scale 5), the short-fitted slope of scale 1–5 (slope 5), the area under MSE scale 1–5 (area 1–5), and the area under MSE scale 6–20 (area 6–20) (Fig. 1). The short-term scales of normal sinus beats may be regulated by respiratory sinus arrhythmia (RSA) and modulated by the parasympathetic system. Area 1–5 is used to estimate the short-term complexity in a system, also called short-term complexity index (CI_s_), attributed to mainly the parasympathetic system, and area 6–20 estimates the overall long-term complexity, also called long-term complexity index (CI_l_). Slope 1–5 describes the dynamic pattern of heart rate, and can serve as a supplementary parameter to complement the information provided by the area under the curve from scales 1 to 5. Specifically, the slope 1–5 offer a measure of how frequent the respiratory triggered the related oscillation within the RR series, independent of the RSA amplitude^31^. In contrast, certain other short-term nonlinear parameters, like MSE area 1–5 or DFAα1, exhibit higher sensitivity to changes in RSA amplitude. Slope 1–5 is typically positive in healthy individuals. However, it may turn negative during the hyperactivation of the sympathetic system or the withdrawal of the parasympathetic system in patients with heart failure^5,32^ and critical illnesses^33^.

Summation of the entropy over different scales can quantify the complexity over certain timescales. Four parameters were calculated: (1) Slope 1–5: linear-fitted slope between scales 1–5; (2) The value of SampEn at scale 5; (3) The area under the curve between scale 1–5 (area 1–5) was used to represent the complexity between short scales, also called short-term complexity index (CI_s_); (4) The area under the curve between scale 6–20 (area 6–20) was used to represent complexity between long scales, also called long-term complexity index (CI_l_).

### Laboratory analysis

All of the patients and controls underwent blood sampling for serum biomarker analysis. The blood samples were placed into a sodium citrate-containing tube. After centrifugation, plasma was collected and stored at − 80 °C until analysis.

### Statistical analysis

The echocardiogram measurements and clinical continuous variables were expressed as mean ± standard deviation based on an assumption of normal distribution. Clinical categorial variables were expressed as absolute and relative percentage. HRV/HRC metrics were expressed as median (25th and 75th percentiles) for non-normally distributed data. The HRV/HRC indices and clinical data were compared using the Mann–Whitney *U* test and Fisher’s exact test, respectively. To compare HRV/HRC metrics at 1 year postinfarct between those with preserved and impaired LV systolic function, we used logistic regression models, and compared the area under the receiver operating characteristic (ROC) curve (AUC) for each HRV/HRC metric. We used net reclassification index (NRI) and integrated discrimination index (IDI) to evaluate the incremental predictive performances by adding a single metric into the original model^34–36^. We added a single HRC metric into a traditional HRV metric model to increase the predictive ability of postinfarct LV systolic impairment. The significance of NRI/IDI was evaluated using normal approximation. All statistical analysis were calculated using IBM SPSS version 26 (SPSS Inc., Chicago, IL, USA). The NRI/IDI models were calculated using R software 4.0.3 (R Foundation for Statistical Computing, Vienna, Austria, http://www.r-project.org, accessed on 10 October 2020). The test of significance was set at 0.05 (p-value < 0.05).

### Ethical approval

The study was approved by the Institutional Review Boards at National Taiwan University Hospital, Taiwan (IRB No: NTUH 104-S2696, UN103-065). Informed consent was obtained from all participants.

## Results

### Clinical characteristics

The clinical and serum biomarker data of the inferior STEMI and control groups are listed in Table 1. Compared to the control group, the inferior STEMI group had higher fasting glucose, higher low-density lipoprotein (LDL) and lower high-density lipoprotein (HDL). None of the STEMI patients died within 1 year of follow-up.

**Table 1:** Clinical data of the inferior STEMI in acute phase and control subjects.

	Inferior STEMI (N = 34)	Control (N = 68)	*p* value
Age (years)	57.5 ± 12.6	57.0 ± 12.3	0.839
Male, n (%)	29 (85.3%)	61 (82.4%)	0.711
DM, n (%)	9 (26.5%)	14 (20.6%)	0.503
Hypertension, n (%)	18 (52.9%)	43 (63.2%)	0.318
Fasting glucose, mg/dL	139.0 ± 67.0	104.3 ± 20.8	**0.001**
Creatinine, mg/dL	1.2 ± 1.2	1.0 ± 0.7	0.315
Triglyceride, mg/dL	129.4 ± 85.5	146.8 ± 90.7	0.359
Total cholesterol, mg/dL	183.4 ± 52.2	167.6 ± 37.4	0.084
LDL, mg/dL	113.5 ± 31.7	91.0 ± 33.0	**0.004**
HDL, mg/dL	36.3 ± 8.9	48.1 ± 11.8	** < 0.001**
Peak CK, U/L	2316 (2968.5)	–	–
Peak CK-MB, U/L	173.1 (174.5–3107.5)	–	–

*DM* diabetes mellitus; *TG* triglyceride; *LDL* low density lipoprotein; *HDL* high density lipoprotein; *CK* creatine kinase; *CK*-*MB* creatine kinase myocardial band.

Significant values are in bold.

### Baseline and serial postinfarct echocardiography

The echocardiogram measurements in both groups are listed in Table 2. The left end-diastolic diameter (LVEDD) and left end-systolic diameter (LVESD) both gradually increased within 1 year postinfarct. In addition, the LVEF recovered within 3 months postinfarct, but remained depressed in the inferior STEMI group compared to the control group. LVESD was consistently higher in the inferior STEMI group than in the control group during follow-up. In addition, LVEDD progressively enlarged in the inferior STEMI group during follow-up, and was significantly larger than in the control group at 1 year postinfarct.

**Table 2:** Baseline and serial postinfarct echocardiogram parameters.

	Echocardiogram				
	Control	Inferior STEMI			
		Acute stage (N = 34)	3 months (N = 20)	6 months (N = 22)	1 year (N = 32)
IVST, mm	11.3 ± 1.5	10.9 ± 1.6	10.6 ± 1.3	10.9 ± 1.3	11.2 ± 2.1
P value*	–	0.257	0.054	0.244	0.793
P value^#^	–	–	0.242	0.832	0.251
LVPWT, mm	10.6 ± 1.9	10.1 ± 1.4	10.5 ± 1.9	10.7 ± 1.2	11.0 ± 1.5
P value*	–	0.697	0.910	0.919	0.350
P value^#^	–	–	0.709	0.383	**0.030**
LVEDD, mm	48.0 ± 4.1	47.1 ± 6.1	49.3 ± 4.6	49.5 ± 4.3	50.4 ± 4.9
P value*	–	0.445	0.228	0.133	**0.014**
P value^#^	–	–	**0.001**	** < 0.001**	**0.004**
LVESD, mm	29.1 ± 4.1	32.8 ± 6.1	32.5 ± 5.5	32.0 ± 4.7	33.7 ± 5.8
P value*	–	**0.003**	**0.005**	**0.008**	** < 0.001**
P value^#^	–	–	0.171	**0.015**	0.182
LVEF, %	69.1 ± 6.4	56.9 ± 10.6	61.5 ± 9.1	62.9 ± 9.0	60.0 ± 9.9
P value*	–	** < 0.001**	** < 0.001**	**0.001**	** < 0.001**
P value^#^	–	–	**0.029**	**0.023**	0.177
EA	0.9 ± 0.3	1.1 ± 0.3	1.0 ± 0.4	1.0 ± 0.2	1.1 ± 0.4
P value*	–	**0.010**	0.234	0.381	**0.039**
P value^#^	–	–	0.409	0.213	0.942

*Using student *T* test compared with control subjects,

^#^Using paired *T* test, compared with inferior STEMI acute stage data.

*IVSD* intraventricular septum diameter, *LVPWD* LV posterior wall diameter, *LVEDD* LV end-diastolic diameter, *LVESD* LV end-systolic diameter, *LVEF* LV ejection fraction.

Significant values are in bold.

### Baseline and serial postinfarct HRV and HRC metrics

The HRV and HRC metrics in the inferior STEMI and control groups are listed in Table 3. The postinfarct HRV metrics showed significantly lower SDNN and higher LF at acute stage and during follow up. The LF/HF ratio increased from 3 months postinfarct in the inferior STEMI group compared to the control group.

**Table 3:** Baseline and serial postinfarct HRV and HRC metrics.

	HRV and HRC metrics				
	Control	Inferior STEMI			
		Acute stage (N = 33)	3 months (N = 20)	6 months (N = 22)	1 year (N = 31)
Mean NN (ms)	793.6 (711.7–903.5)	823.6 (691.5–882.9)	725.1 (672.3–801.1)	753.1 (716.7–827.5)	720.9 (689.1–820.1)
P value*	–	0.338	**0.009**	0.124	**0.046**
P value^#^	–	–	**0.041**	0.585	0.366
SDNN (ms)	73.5 (58.2–89.2)	44.2 (27.9–50.3)	36.8 (29.8–44.8)	35.2 (30.4–40.8)	36.0 (24.9–46.0)
P value*	–	** < 0.001**	** < 0.001**	** < 0.001**	** < 0.001**
P value^#^	–	–	0.601	0.636	0.518
pNN20 (%)	21.1 (12.1–39.0)	24.0 (16.4–38.8)	23.8 (14.6–32.7)	21.3 (14.8–31.0)	18.4 (12.0–32.2)
P value*		0.822	0.895	0.470	0.680
P value^#^	–	–	0.356	0.167	0.331
pNN50 (%)	2.4 (0.8–8.0)	2.1 (0.7–7.5)	2.9 (1.0–4.7)	1.6 (0.9–2.9)	1.7 (0.7–3.8)
P value*	–	0.425	0.143	0.080	0.247
P value^#^	–	–	0.170	0.086	0.446
LF (ms^2^)	39.2 (16.8–65.6)	198.2 (71.8–347.3)	149.3 (54.1–213.8)	119.1 (57.1–213.2)	126.7 (60.5–178.8)
P value*	–	** < 0.001**	** < 0.001**	** < 0.001**	** < 0.001**
P value^#^	–	–	0.231	0.219	0.124
HF (ms^2^)	14.6 (5.1–28.4)	56.3 (20.6–131.0)	27.9 (19.4–54.8)	25.6 (18.8–45.8)	23.7 (15.8–51.0)
P value*	–	**0.005**	0.102	0.135	**0.024**
P value^#^	–	–	0.087	0.097	0.156
LF/HF	2.8 (1.2–4.7)	3.9 (2.4–5.7)	4.1 (3.3–6.7)	5.1 (2.8–7.1)	4.7 (3.0–7.0)
P value*	–	0.086	**0.004**	**0.002**	**0.003**
P value^#^	–	–	0.448	0.354	0.343
DFAα1	1.2 (0.9–1.3)	1.2 (1.0–1.3)	1.2 (1.0–1.3)	1.2 (1.0–1.4)	1.2 (1.1–1.3)
P value*	–	0.929	0.310	0.159	0.325
P value^#^	–	–	0.327	0.090	0.182
DFAα2	1.1 (1.1–1.2)	1.0 (1.0–1.1)	1.1 (1.0–1.2)	1.1 (1.1–1.1)	1.1 (1.1–1.2)
P value*	–	** < 0.001**	0.244	0.350	0.307
P value^#^	–	–	** < 0.001**	** < 0.001**	** < 0.001**
MSE slope 1–5	0.044 (– 0.018–0.081)	0.042 (− 0.009–0.074)	0.048 (-0.020–0.083)	0.054 (-0.008–0.087)	0.031 (0.009–0.079)
P value*	–	0.944	0.677	0.503	0.561
P value^#^	–	–	0.809	0.421	0.497
Scale5	1.2 (1.1–1.4)	1.5 (1.2–1.6)	1.3 (1.0–1.5)	1.3 (1.1–1.4)	1.2 (1.0–1.5)
P value*	–	**0.032**	0.766	0.905	0.601
P value^#^	–	–	**0.022**	**0.021**	**0.004**
Area 1–5	6.0 (5.1–6.6)	5.2 (4.4–5.9)	4.6 (3.6–5.3)	4.7 (4.2–5.4)	4.4 (3.8–5.3)
P value*	–	**0.003**	** < 0.001**	** < 0.001**	** < 0.001**
P value^#^	–	–	**0.006**	**0.014**	** < 0.001**
Area 6–20	20.5 (18.7–22.6)	22.4 (17.6–24.1)	20.7 (16.5–22.3)	21.2 (18.8–22.6)	19.6 (17.9–22.9)
P value*	–	0.429	0.186	0.441	0.168
P value^#^	–	–	**0.036**	0.098	**0.010**

*Using student *T* test compared with control subjects.

^#^Using paired *T* test, compared with inferior STEMI acute stage data.

*SDRR* standard deviation of normal heartbeats, *pNN*_*20*_ percentage of the successive change in RR interval exceeds 20 ms, *pNN*_*50*_ percentage of the successive change in RR interval exceeds 50 ms, *LF* low frequency, *HF* high frequency, *DFA* detrended fluctuation analysis, *MSE* multiscale entropy, *slope 1–5* slope between scale 1–5, *Area 1–5* the area under scale 1–5, *Area 6–20* the area under scale 6–20.

Significant values are in bold.

In HRC analysis, DFAα2 was consistently depressed from the acute event to 1 year postinfarct. Compared to the value of scale 5 during the acute event, postinfarct scale 5 progressively decreased, but there was no significant difference in scale 5 between the control and inferior STEMI groups. The short-term complexity index was consistently depressed in the inferior STEMI group compared to the control group, and progressively declined until 1 year postinfarct. There were no significant differences in long-term complexity indices between the inferior STEMI and control groups.

### HRV and HRC metrics to predict postinfarct LV systolic impairment

**Figure 2:**
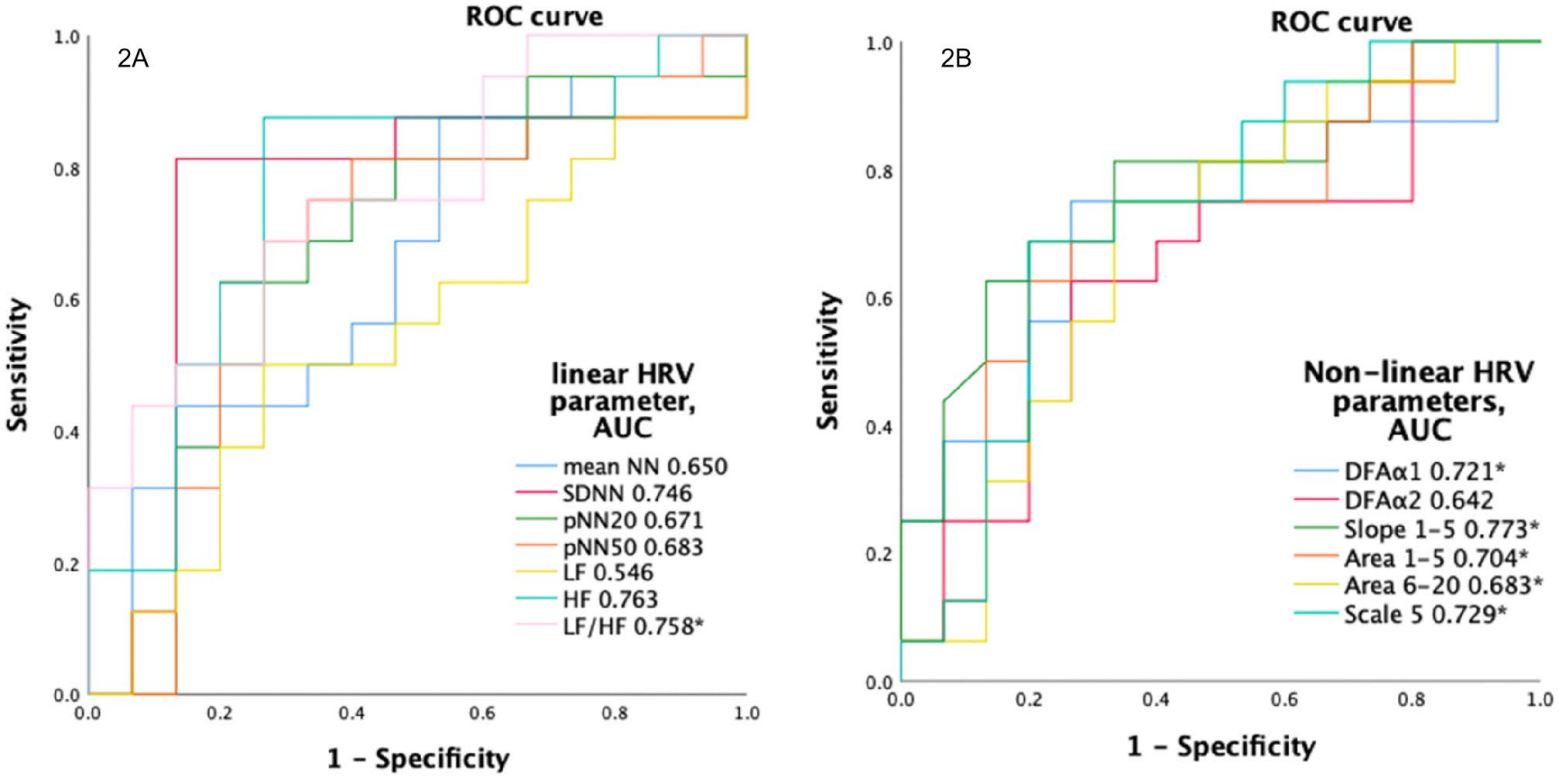
The ROC curves of HRV/HRC metrics ((**2A**) HRV metrics, (**2B**) HRC metrics) at 1 year postinfarct to predict LV systolic impairment. *SDRR* standard deviation of normal heartbeats; *pNN20* percentage of the successive change in RR interval exceeds 20 ms; *pNN50* percentage of the successive change in RR interval exceeds 50 ms; *LF* low frequency; *HF* high frequency; *DFA* detrended fluctuation analysis; *slope 1–5* slope between scale 1–5; *Area 1–5* area under MSE scale 1–5; *Area 6–20* area under MSE scale 6–20; *p < 0.05.

The median LVEF at 1 year postinfarct was 59%. We then categorized the inferior STEMI patients into two subgroups based on the median LVEF at 1 year postinfarct. Eighteen patients were enrolled in the preserved LV systolic function group, and the other 16 in the depressed LV systolic function group. The median LVEF values in the with and without LV systolic impairment groups were 54.2% and 66.0%, respectively. ROC curves of HRV and HRC metrics at 1 year postinfarct were plotted to predict LV systolic impairment (Fig. 2). In HRV metrics, HF, LF/HF ratio and SDNN all had high AUC values (HF AUC 0.763, SDNN AUC 0.746, LF/HF ratio AUC 0.758). In HRC metrics, slope 1–5 had an AUC value of 0.773, the highest among all metrics. Slope 1–5 had the best predictive power for postinfarct LV systolic impairment among all HRV and HRC metrics.

### Adding slope 1–5 to predict postinfarct LV systolic impairment using NRI/IDI

We then added slope 1–5 in linear metrics using NRI/IDI models to differentiate postinfarct LV systolic impairment. The values of the NRI/IDI models are listed in Table 4). After adding slope 1–5, pNN20, pNN50, HF, and LF/HF ratio increased the predictive power when combined with slope 1–5 in the model.

**Table 4:** AUC, NRI, and IDI models of linear parameters before and after adding HRC metrics (slope 1–5) to predict LV systolic impairment at 1-year postinfarct.

				Continuous NRI/IDI			
Parameters	AUC	P value	R-square	NRI	NRI p-value	IDI	IDI p-value
SDNN	0.704		0.117				
+ Slope1-5	0.77	0.351	0.197	0.533	0.113	0.083	0.087
pNN20	0.63		0.031				
+ Slope1-5	0.763	0.239	0.201	0.911	**0.003**	0.176	**0.01**
pNN50	0.567		0.001				
+ Slope1-5	0.778	0.118	0.172	1.044	** < 0.001**	0.179	**0.008**
LF	0.77		0.086				
+ Slope1-5	0.763	0.914	0.195	0.533	0.113	0.098	0.062
HF	0.619		0.047				
+ Slope1-5	0.767	0.208	0.182	0.8	**0.012**	0.143	**0.018**
LF/HF	0.663		0.01				
+ Slope1-5	0.778	0.339	0.182	0.689	**0.033**	0.187	**0.007**

*SDRR* standard deviation of normal heartbeats, *pNN*_*20*_ percentage of the successive change in RR interval exceeds 20 ms, *pNN*_*50*_ percentage of the successive change in RR interval exceeds 50 ms, *LF* low frequency, *HF* high frequency, *slope 1–5* slope between scale 1–5.

Significant values are in bold.

## Discussion

The main finding of this study is that many linear HRV and HRC variables were depressed in inferior STEMI patients during follow-up after infarction. In addition, among all HRV and HRC variables, slope 1–5 had the best discriminatory power to predict postinfarct LV systolic impairment. Furthermore, slope 1–5 significantly improved the predictive power of the linear HRV variables for postinfarct LV systolic impairment.

In our previous investigation, we found that postinfarct HRV and HRC metrics were depressed in anterior STEMI patients^11^, presenting as a consistently depressed slope 1–5, decreased area under scale 1–5 (area 1–5), and decreased area under scale 6–20 (area 6–20) during follow-up. Similar to these findings in anterior STEMI patients, the HRC results in the present study showed a gradual decrease in scale 5, decrease in area 1–5 and decrease in area 6–20 (but without significance at 6 months postinfarct) after the acute event in inferior STEMI patients. Both short-term and long-term complexity indices had a similar declining pattern during 1 year of follow-up after the acute stage in anterior and inferior STEMI patients. Few HRV studies focused on inferior STEMI because of the possible effect of BJ reflex. Anterior and inferior STEMI had different impaired HRV pattern within 2 days postinfarct. Despite that, the SDNN tend to recover early at 2-week postinfarct in both anterior and inferior STEMI^37^. In the present study, we focused on inferior STEMI without clinically significant BJ reflex, and the main results confirmed a similar depressed postinfarct HRC pattern to patients with anterior STEMI in a long time scale.

Sympathetic activation may result in reducing short-term complexity^38^ and HRV parameters^39^. Sympathetic activation may impact short time scale and sympathetic modulation may impact in long time scale^40^. The postinfarct sympathetic activation and LV remodeling may occurred quickly^41,42^, as early as within 72 hours^43^. The RAS activation contribute sympathetic activation in LV remodeling process. Hyperactive RAS had a reversible impact in depressed HRV and HRC as well^44^. Sympathetic activation had a potential role in causing lower SDNN, higher LF power, higher LF/HF ratio and lower MSE area 1–5 in inferior STEMI. The long time scale complexity in STEMI also had a trend of decrease in our study. Both LF power, and LF/HF ratio showed a trend of slowly recovery in postinfarct follow up, which may be explained by the gradually improvement of sympathetic activation and the beta-blocker prescription in standard care. Beta-blocker reduced postinfarct mortality and was recommended to use as long as tolerance in standard postinfarct care by guidelines^45^. Beta-blocker may improve HRV^46^ and MSE parameters^31^, causing recovery of lower LF, and decreased entropy in short time scale. Most STEMI patients will receive at least low dose beta-blocker based on guideline^45^. However, the prescription and tolerance status of beta-blocker in this study was not available.

The cardiovascular control of heartbeat was complicated, and modulated by sympathetic, vagal innervation, neurohormone system, blood pressure change, sinus respiratory coupling, postural change, and exercise intensity^31,40,47–50^. The modulation of sympathetic and baroreflex had a different pattern of contribution in linear HRV and non-linear complexity analysis and impacted differently in short time scale and long time scale^40^. However, the exact physiologic role of every single MSE parameters in STEMI was still unclear. The slope between scale 1–5 showed different patterns in different pathological condition. In health subjects, the slope between scale 1–5 was mostly positive, whereas it turned to be negative in heart failure^28^. Negative slope between scale 1–5 was also observed in other pathological conditions^51^.

Factors associated with postinfarct mortality include decreased LV contractility, arrhythmia, depressed HRC/HRV variables and inferior STEMI^13,52–55^. In the current era of primary percutaneous coronary intervention with improved techniques and advanced equipment, STEMI-associated mortality has decreased to less than 7.8% at 30 days^2^. None of the inferior STEMI patients died during 1 year of follow-up in the present study. Therefore, using a surrogate prognostic marker is important. Since postinfarct LV contractility was associated with survival, the serial change in postinfarct LV contractility during follow-up could be used as a surrogate marker. Previous studies have demonstrated the favorable prognostic value of postinfarct HRV variables, including higher LF, SDNN, and DFAα1^11,13,56^. However, the association between HRV/HRC and postinfarct LV systolic impairment is uncertain. This is the first study to investigate the association between postinfarct LV systolic impairment and HRV/HRC. Serial postinfarct echocardiograms during follow-up showed progressively enlarged LVEDD and LVESD. The LVEF at 3 months postinfarct recovered, but its value remained significantly lower than in the control subjects at 1-year postinfarct. Serial MSE curves at different time scales during follow-up showed a similar temporal pattern of decline in LVEDD and LVESD.

Compared to the patients without postinfarct LV systolic impairment at 1-year follow up, the patients with postinfarct systolic impairment at 1-year follow up had more depressed initial HRV/HRC variables at acute stage, including lower SDNN (28.8 vs 46.7, p = 0.047), lower LF value (77.1 vs 298.8, p = 0.008) and lower MSE slope 1–5 (0.005 vs 0.06, p = 0.008). The prognostic value of higher SDNN and higher LF for postinfarct survival have been investigated before^13,55^, however this is the first study to show that patients with postinfarct LV systolic impairment also had a low SDNN and low LF.

In ROC analysis, slope 1–5 had the best discriminative power (AUC 0.773) in predicting postinfarct LV systolic impairment among all HRV/HRC variables. In addition, in the NDI/IDI prediction models, adding slope 1–5 improved the predictive power of linear HRV measurements in predicting postinfarct LV systolic impairment, including a lower SDNN and higher LF. This is the first study to report the predictive ability of slope 1–5 for postinfarct systolic impairment. Slope 1–5 has been used to describe the short-term complexity pattern, and it has been reported to be significantly depressed in diseased patients, including those with pulmonary arterial hypertension and heart failure^9,11,32,57^. The hypothesis of slope 1–5 is based on stronger respiratory sinus coupling in a short-term scale in healthy subjects^5^, which is mainly due to baroreceptors. In our previous investigation, we found a decreased slope 1–5 in post-anterior STEMI patients^11^, and that higher scale 5 and higher area 1–5 were associated with better outcomes in cardiovascular diseases^9,58,59^. As in our previous investigation, HRC indices had a better diagnostic and prognostic power compared to traditional HRV indices in the present study.

Traditionally, echocardiograms have proven invaluable in assessing left ventricular contractility following an infarction, primarily within medical center settings. Unfortunately, these echocardiographic capabilities have not been readily accessible to physicians in many local clinics. In contrast, the utilization of ambulatory Holter recordings offers an alternative avenue for physicians to assess post-infarct heart rhythm alteration^60^. Its potential significance lies in its accessibility and ease of use, as it might not require specialized medical personnel for signal analysis. This aspect makes Holter monitoring particularly appealing for applications such as remote healthcare and underserved rural areas. Through the analysis of Holter signals, a broader spectrum of information beyond rhythm fluctuations becomes available, enhancing the diagnostic capabilities for evaluating cardiac health.

There are several limitations to this study. First, this is a pilot study with a small sample size, and further larger clinical studies are needed to confirm our findings. Secondly, it is important to acknowledge that potential confounding factors may still exist between inferior STEMI patients and the control group, which could potentially interfere with the observed differences in HRV parameters in this study. Additionally, the selection of preprocessing methods for the original RR intervals or complexity analysis can potentially compromise the sensitivity of the resultant parameters. Therefore, the study's results should be interpreted with cautious consideration of these confounding influences. Third, during the initial episode, most inferior STEMI patients are strictly restricted to bed before cardiopulmonary rehabilitation, which may contribute to confounding factors for HRV such as posture change and exercise^61^. Fourth, none of the inferior STEMI patients died within 1 year of follow-up. Therefore, we could not analyze differences in HRV parameters between survivors and non-survivors. Fifth, the medication use and adherence which could be the potential confounders were unavailable in this study.

## Conclusion

Postinfarct HRV and HRC were depressed in the inferior STEMI patients, and MSE slope 1–5 had good predictive power for postinfarct LV systolic impairment.

## Data Availability

The datasets used and analyzed during the current study available from the corresponding author on reasonable request.
